# Which intervention synergies maximize AGYW’s HIV outcomes? A classification and regression tree analysis of layered HIV prevention programming

**DOI:** 10.1097/QAI.0000000000003289

**Published:** 2023-12-01

**Authors:** Sanyukta Mathur, Bidhubhusan Mahapatra, Raman Mishra, Craig J. Heck, Michael Mbizvo

**Affiliations:** 1. Population Council, Washington, D.C.; 2. Norwegian Refugee Council, Amman, Jordan; 3. College of Health Science, Korea University, Seoul, South Korea; 4. Department of Epidemiology, Columbia University Mailman School of Public Health, New York, NY, USA; 5. Population Council, Lusaka, Zambia

**Keywords:** AGYW, HIV prevention, classification and regression trees, multi-sectoral programs, combination prevention, Zambia

## Abstract

**Introduction:**

Intersecting behavioral, social, and structural factors increase adolescent girls’ (AG) and young women’s (YW) HIV vulnerability. Yet, understanding of optimal intervention synergies remains limited. We identified intervention combinations that statistically maximized reductions in AGYW’s HIV-related risk.

**Methods:**

Using data collected in 2018 with Zambian AG (n=487, aged 15–19 years) and YW (n=505, aged 20–25 years) after 12–14 months exposure to DREAMS (multi-sectoral HIV program), we used classification and regression trees to explore relationships between interventions (safe space/social asset building [SAB] and provision of/linkage to youth-friendly health services [YFHS], education social protection [Educ], economic social protection [Econ]) and HIV-related outcomes (HIV testing, consistent condom use, transactional sex, sexual violence experience from partners and non-partners).

**Results:**

Overall, 59.9% completed SAB and 81.5%, 35.4%, and 29.6% received YHFS, Educ, and Econ, respectively. For AG, HIV testing improved (73%->83%) with exposure to all interventions, condom use improved with Econ (33%->46%), transactional sex reduced with SAB+Educ, and sexual violence from partners and non-partners reduced with Educ and SAB, respectively. For YW, HIV testing increased with Educ (77%->91%), condom use increased with SAB+YFHS (36%->52%), transactional sex reduced with combinations of all interventions, and sexual violence from partners reduced with YFHS and from non-partners with SAB+Econ.

**Conclusions:**

Tailored interventions might be more effective than uniform combination intervention packages in reducing AGYW’s HIV risk. AG benefitted most from SAB and/or Educ, while YFHS, Educ, and/or SAB reduced YW’s HIV-related risk. Educational and asset-building interventions could have the greatest impact on AGYW’s HIV risk.

## Introduction

The achievement of HIV epidemic control remains a priority in eastern and southern Africa, which accounts for approximately 38% of global HIV incidence.^1^ Adolescent girls and young women (AGYW, ages 15–24 years) in sub-Saharan Africa account for over 77% of all new infections among AGYW globally, partly due to intersecting behavioural, biological, and structural risk factors.^1^ To address this need, the DREAMS partnership developed and implemented a comprehensive, multi-sectoral HIV prevention package to reduce HIV risk and improve HIV-related outcomes for AGYW. The DREAMS approach extended beyond HIV and youth-friendly sexual and reproductive health services to include social asset building and key social protection interventions (e.g., educational subsidies).^2^ This collection of interventions aimed to empower AGYW with the necessary skills and resources to reduce their HIV vulnerability and risky sexual behaviors and increase their access and use of healthcare services, all within an enabling environment. Since its inception in 2015, similar comprehensive, multi-sectoral HIV prevention approaches are also being considered by other multi-lateral and national entities.^3,4^

However, there is insufficient knowledge of how these combination HIV prevention interventions influence AGYW’s HIV-related outcomes.^5^ In theory, the concurrent receipt of multiple HIV prevention interventions within a specified timeframe (i.e., layering) should result in synergies that maximize their effectiveness.^6^ Without evidence corroborating this hypothesis, programmers and policymakers do not have clear guidance on the optimal combination of interventions required to achieve the desired effects. Randomized control trials have delineated the efficacy of specific HIV prevention interventions, yet these studies are narrow in scope and offer little insight into the effectiveness of interventions that are implemented at scale.^7^ In program evaluation literature, use of traditional methods like, multivariable modeling approaches often inadequately account for interactions between exposures of interest, potential biases, and other covariates, producing unreliable results.^8^ Alternative novel analytical approaches need to be applied to assist organizations in making the best programmatic decisions to reduce HIV risk among AGYW.

In this study, we use classification and regression tree (CART) analysis to unearth complex interactions between exposure to Determined, Resilient, Empowered, AIDS-free, Mentored, Safe (DREAMS) interventions and the desired outcomes.^9–12^ Due to DREAMS’ multi-component, -level, and -sectoral programming approach, CART analysis offers a unique opportunity to examine the effects of layered HIV prevention programming on HIV-related outcomes, including HIV testing, transactional sex, consistent condom use, and experience of sexual violence. Per the DREAMS program logic model,^2^ these program outcomes are key to reducing AGYW’s HIV risk. Among the DREAMS program components (social asset building [SAB], youth friendly health services [YFHS], education social protection [Educ], and economic social protection [Econ]), we examine which combinations influence the likelihood of HIV-related outcomes among urban AG YW in Zambia. We stratify our analysis by age group due to increasing risk of HIV acquisition by age and to elucidate program components that might be most impactful in reducing HIV risk for younger versus older participants.^13^

## Methods

### Intervention description

The framework, approach, and rationale for DREAMS’ multi-country program is described elsewhere.^2^ In brief, at each DREAMS program site, implementing partners (IP) identify AG and YW to participate in the program. In each country context, a primary package of interventions was defined, with the expectation that all DREAMS participants would receive the primary package and additional interventions depending on individual need or vulnerability.

The foundation of Zambia’s primary package includes SAB, delivered through a safe space platform. At these safe spaces, AGYW socialize with peers and mentors, participate in facilitated instruction on health and life skills focused on HIV prevention-related topics (e.g., violence prevention/consent, puberty etc.), receive youth-friendly health services (e.g., access to HIV testing services and condom distribution, linkages to contraceptives, PrEP, post-violence care, as needed), and educational or economic social protection interventions (e.g., support for school fees/expenses, economic subsidies, participation in savings groups).^14^

### Evaluation data and procedures

We used data from quantitative surveys collected from DREAMS enrollees, AG (aged 15–19, n=380) and YW (aged 20–25 years, n=505), in urban Lusaka and Ndola, Zambia between March-May 2018. These respondents were previously interviewed in 2016–2017 upon enrolling into a DREAMS program evaluation cohort. This analysis includes data from enrolled AGYW that we were able to re-interview after 12–14 months of DREAMS exposure (83.2% of the original sample).^15^ Although the program and research team made repeated attempted to re-contact interviewees, the most common reason for attrition was extended or permanent out-migration from the study communities.

Female data collectors conducted in-person, one-on-one interviews in private, convenient locations (e.g., room in respondent’s home, nearby field, or nearby community center) and out of earshot of parents, guardians, or other community members. The structured survey captured information on knowledge, attitudes, practices, program exposure and HIV service uptake in the local language (Bemba and Nyanja).

### Measures

We examined exposure to four key DREAMS interventions: SAB, YFHS, Educ, and Econ (Table 1). We examined five outcomes that are important for HIV prevention among AGYW: in the last 12 months, HIV testing, transactional sex engagement, consistent condom use, and sexual violence from an intimate or non-intimate partner.

### Statistical Analysis

We conducted descriptive and CART analysis. Descriptive analysis described the study participant profiles. We performed CART analysis to identify program intervention pathways that statistically maximize gains in program outcomes. CART analysis is a predictive modelling technique that uses non-parametric classification approach to recursively partition the data by introducing one predictor variable at a time to create two mutually exclusive, homogenous data subgroups.^12,16^ Splits are created to minimize within-node differences while maximizing between-node variances. The partitioning process continues until all possible splits are exhausted or a pre-defined stopping criterion is reached. A tree’s terminal node captures the maximum likelihood for an outcome based on all the branches and splits leading up to it. Ultimately, the goal of CART analysis is to sub-divide data into mutually exclusive, collectively exhaustive groupings to increase the management and interpretability of underlying synergies.^17^ This approach has been used in other papers,^18–20^ which describe the relative advantage of CART over multivariable regression. The main distinction between more conventional methods (e.g., multivariable regression) and CARTs is the treatment of interactions in the models. Conventional methods assume that predictors act independently, and while interactions can be modeled, they are usually omitted to reduce complexity and promote parsimony. In contrast, CART assumes interactions are the rule rather than the exception, allowing this analysis to model multilevel interactions that would be laborious, if not impractical, using traditional regression analyses.^21^ We performed all CART analyses using PASW Statistics (Version 18). We present the full CART models (Supplemental Figures 1-5) as well as a summary of the optimized pathways indicating percentage improvement in prevalence of an outcome attributed to the pathway. Further, we examined the characteristics of respondents in each pathway to assess how these respondents compared with the overall study population.

### Ethics

We obtained written informed consent from adult participants (i.e., those above the age of consent) before each round of data collection. If the participant was a minor, we obtained parental consent and participant assent. The study protocols were reviewed and approved by Population Council’s Institutional Review Board (Protocol #744) and ERES Converge Institutional Review Board and National Health Research Authority in Zambia (2016-May-016). Participants were compensated 50 Zambian Kwacha (~USD $5 at the time of the study) for their time, per local ethical guidelines. Participation in this study did not influence AGYW’s access to or participation in any DREAMS interventions. We engaged study communities and IPs throughout the research process. During study planning, we sought approval from community leadership and refined research questions and tools with IPs. We also held dissemination meetings to share findings, study outputs, and recommendations with community leaders, IPs, and key stakeholders and decision-makers.

## Results

### Profile of participants

A little more than half of the participants were YW (20–25 years old) at the time of the survey (Table 2). AGYW differed significantly in their profile when examined by age group. When compared with AG, orphanhood (45.0% vs. 36.1%) and travel outside the community (77.8% vs. 72.1%) were higher among YW. Alternatively, more AG were working (49.2% vs. 41.8%) and in school (66.3% vs 49.7%) than YW. Exposure to various program components also differed by age-group. Particularly, a smaller proportion of YW received YFHS (77.6% vs 86.6%) and Educ (25.1% vs 48.9%). While over 90% had accessed SAB, ~60% overall had completed the SAB intervention. Econ exposure (~30% overall) was also similar across both age-groups.

### CART results

CART analyses suggest that the four program variables, either alone or in combination, optimized the outcomes of various HIV-related measures. Table 3 presents a summary of the CART analysis results for AG and YW. In reading Table 3, “Yes” indicates receipt of the intervention component, “No” means they did not, and a blank cell indicates that the intervention component did not feature in any of the optimal pathways in the CART model. The blanks arise when individuals who received the intervention versus not do not behave differently.

#### HIV Testing

The proportion of HIV testing among AG is likely to increase by 15% from the base value of 73% if they are exposed to all intervention components. (Table 3). Supplemental Figure 1A shows the full model. The node at the very top indicates the overall occurrence of this outcome among adolescent girls. Specifically, 73% of all adolescent girls tested for HIV in the last 12 months. With each split from the source node to the terminal node, we see an improvement in this proportion. For example, 76% of adolescent girls who received youth friendly health services had tested for HIV in the last 12 months. This proportion increases to 79% among adolescent girls who received youth friendly health services and completed social asset building. Among adolescent girls who completed social asset building and received youth friendly health services and economic social protection exposure, the proportion who tested for HIV was 81%. Finally, adolescent girls who received all the prior interventions plus educational social protection had the highest occurrence of HIV testing in the last 12 months: 83%. The goal for interpreting these trees is to identify which pathway yields the greatest increase from the base value (i.e., overall proportion among the sample). We explored the characteristics of AG in this pathway to provide additional information on who should be reached with these interventions. A majority of the AG had travelled outside the community and were currently in school, bout one-quarter of AG within this pathway had been orphaned and15% were engaged in paid work. (Supplemental Table 1).

YW who received Educ but not SAB and Econ had 18.4% higher HIV testing from the base value of 77% (Table 3, Supplemental Figure 1B). More than one-half of YW within this pathway were orphans, were engaged in paid work, had ever travelled outside of the community, and very few (9%) were still in school (Supplemental Table 1).

#### Consistent Condom Use

Among AG, those exposed to Econ had 39% higher likelihood of using condoms consistently from the base value of 33% (Table 3, Supplemental Figure 2A). About one-third of the AG within this pathway were orphans, about one-fifth were engaged in paid work, and approximately two-thirds had ever travelled outside of the community or were currently enrolled in school (Supplemental Table 1).

For YW, exposure to SAB and YFHS but not Educ and Econ resulted in a 46.1% increase in consistent condom from the base value of 36% (Table 3, Supplemental Figure 2B). YW in this pathway were defined by high levels of paid work, travel outside the community, and in-school status (Supplemental Table 1).

#### No Transactional Sex

Two intervention pathways completely reduced AG’s engagement in transactional sex (i.e., a 100% outcome proportion): 1) no exposure to SAB or YFHS, and 2) exposure to SAB and Educ (Table 3, Supplemental Figure 3A). About 40% of the AG in the first pathway were orphans, 74% have travelled outside of the community or were in school, and one-third were engaged in paid work. Relative to the first pathway, all metrics in the second pathway were lower (Supplemental Table 1).

Three potential pathways led to a similar outcome proportion among YW: 1) exposure to Econ but not YFHS, 2) exposure to YFHS, Educ, and Econ but not SAB, and 3) exposure to SAB, YFHS, and Educ but not Econ (Table 3, Supplemental Figure 3B). In all three pathways, high proportions of YW were engaged in paid work, travel outside the community was nearly universal, and few were in-school. Pathway one had fewer orphanhood cases than pathways two and three (Supplemental Table 1).

#### No Intimate Partner Sexual Violence

Among AG who received Educ but not Econ, this outcome was statistically maximized by 9% from a base value of 76% (Table 3, Supplemental Figure 4A). AG in this pathway were characterized by orphanhood (40%), high travel outside the community (76%) and in-school status (66%), and about one-third were involved in paid work (26%).

If YW were exposed to YFHS but not SAB and Econ, reports of no intimate partner sexual violence were 7.1% higher from a base value of 82% (Table 3, Supplemental Figure 4B). Among YW, the proportion involved in paid work and travel outside the community was higher than their younger counterparts (Supplemental Table 1).

#### No Non-partner Sexual Violence

If exposed to SAB but none of the other interventions, no AG reported non-partner sexual violence. Among AG, about 40% were orphans, most had travelled outside the community or were currently in school, but only a third were engaged in paid work.

For YW receiving SAB and Educ but not Econ led to a 11% increased likelihood of not experiencing sexual violence from non-partners (Table 3, Supplemental Figures 5A-B). YW in this pathway had high engagement in paid work, were mostly out of school, and half had experienced orphanhood (Supplemental Table 1).

## Discussion

Our study highlights that in communities heavily affected by HIV, a comprehensive package of health and non-health interventions is needed to improve AGYW’s HIV-related outcomes. We find that select combinations of health and non-health interventions work better to improve specific HIV-related risk outcome. Access to educational social protection and social asset building interventions increased the likelihood of improving several HIV-related outcomes among both AG and YW. Access to youth friendly health services was an additionally important intervention for YW, as was access to economic social protection interventions for AG. We also present the socio-demographic profiles of AG and YW in the pathways. These provide additional information on who should be reached with the intervention(s). Our findings pinpoint the need to carefully consider which program inputs are most essential for achieving the desired HIV risk-reduction outcomes among AGYW.

The key strength of this paper is our use of CART models to uncover the combination of interventions that optimizes the achievement in outcomes. By considering, both, the synergistic and additive effect of its inputs, this approach identified which intervention package combination works best for each HIV-related outcome. Additionally, this study adds to the burgeoning literature focused on illuminating optimal combinations of multi-sectoral HIV prevention interventions for AGYW.^22–24^ We are also one of the first to apply this approach to an AGYW’s HIV prevention program in sub-Saharan Africa.^20^ Our approach highlights combinations of interventions that may increase or decrease the likelihood of AGYW’s HIV-related outcomes. These examinations are sorely needed, as to meet global targets for HIV prevention and care, there is growing interest in reaching beyond the health sector to address the health and social determinants of health and HIV-risk, particularly among AGYW. A key challenge for decision-makers is unpacking what permutation of programs successfully reduces risk for AGYW and for what outcomes. Our utilization of the decision tree approach begins to answer some of these questions.

For informing programming decisions, our study reveals the need to delve deeper into the pathways that maximize the likelihood of HIV-protective outcomes. HIV testing/knowing one’s status is key to the HIV prevention cascade. Among AG in our sample, testing increased with exposure to the full DREAMS layered model. This might represent the numerous social, structural, and financial barriers AG face when trying to access and/or use HIV prevention services,^25–27^ which can negatively affect their uptake and use of HIV prevention methods, like condoms and PrEP. For YW, educational social protection was key to increasing testing. DREAMS’ layered approach might have been an effective way to reach AG with testing services outside of health clinics.^28^

Condom use is persistently low among AGYW, primarily due to limited access, unequal power dynamics within the partnership leading to failed condom negotiations, and prevalent norms around condom use and practices.^29–33^ Fortunately, we found exposure to SAB and YFHS increased YW’s likelihood of consistent condom use. SAB activities aimed to build health promotion and disease prevention knowledge and self-confidence regarding condom negotiation, while YFHS sought to connect AGYW to health services with youth-friendly providers. Our findings indicate that knowledge and skill improvements might need to be coupled with referrals to accessible health services to have a positive impact on YW’s condom use. Alternatively, access to economic social protection (such as engagement in savings clubs) was sufficient to increase AG’s likelihood of consistent condom use. This finding aligns with results from Tanzania, where the *Mabinti Tushike Hatamu!* Program—which offered life-skills training, income-generating activities, and GBV education—increased reports of consistent condom use among girls enrolled in the program versus not.^34^ Additional research from the region further reinforces the value of economic assets. Randomized trials in Tanzania and Malawi discovered that cash transfers lowered the incidence and/or prevalence of sexually transmitted infections among girls in the intervention arm.^35,36^ This evidence suggests increased economic assets may have the potential to reduce sexual risk behaviours.

Reports of transactional sex were rare among our sample. Transactional sex is a significant risk factor for HIV acquisition, facilitating condomless sex, violence experiences, and other negative outcomes among AGYW.^37–40^ For AG, SAB and Educ completely reduced the outcome, statistically. We also found one pathway with no exposures yielding complete statistical reduction in transactional sex. While this appears paradoxical, it could be indicative that SAB is the most important split, and if AG are not receiving the complete intervention, adding on any other intervention is not maximizing the outcome. Additionally, there might be other explanatory variables or interventions we did not or could not measure using the survey data. YW benefitted from all the interventions in varying combinations. Economic social protection interventions seem to be the most significant in reducing their transactional sex engagement, which is further statistically maximized by layering on YFHS, SAB, and/or Educ. Our prior research found borderline significant increases in transactional sex among Zambian YW.^15^ It is encouraging to find pathways to dissect this potentially negative finding.

Research globally, and in this study setting, have shown the high rates of sexual violence experienced by AGYW and their effects on their HIV and mental health outcomes.^38,41,42^ For reductions in sexual violence from intimate partners, we find that exposure to single intervention components – Educ for AG and YFHS for YW – statistically maximized the desired outcomes. Despite violence-reduction interventions yielding mixed results,^43–48^ keeping AGYW in school has exhibited protective properties, possibly because it provides AGYW a safe, secure location to congregate during the day.^49^ Educational attainment also improves AGYW’s threat perception and appraisal skills.^50,51^ For YW, access to YFHS may have serve as a secondary prevention (e.g., providing post-GBV care/counselling) or provided other services (e.g., PrEP, family planning)^52,53^ that reduced conflict within intimate partnerships.^54^ For non-partner sexual violence pathways, we find that SAB is key intervention. AG were less likely to experience non-partner sexual violence if they had completed the SAB; YW experienced a similar benefit from SAB, when paired with access to educational social protection. In other words, layering activities to expand social networks and improve awareness of health information with interventions that facilitated staying in school (e.g., school supplies, support for school fees) protected AG and YW from experiences of IPV and non-partner sexual violence, respectively. Expanded social networks might translate to AGYW traveling the community together as a safety measure,^55^ which may dissuade men from perpetrating sexual violence. ‘Together, social cohesion and schooling may offer AGYW safe mobility around communities and to, from, and within protective environments, such as schools.

While we were able to identify optimal intervention combinations to improving specific HIV-related outcomes, there remain several unanswered questions. The CART model does not consider the sequencing or quality of implementation. Our prior research shows that the quality and coordinated implementation of the layered DREAMS approach improved over time.^14^ Additional studies may need to explore the sequencing of interventions. Further reflection may also be needed on specific inputs provided in the social protection interventions. For instance, the Econ interventions did not feature in many pathways. It is possible that inputs were insufficient, the exposure too limited, or that prevailing gender norms limited AGYW’s financial autonomy and agency.^56^ In our urban sample, high proportions of AGYW were in school; it is possible the small infusion of educational support offered by DREAMS propelled school continuation among AGYW.^15^ Finally, any AG pathway that featured Educ social protection, respondent characteristics displayed the majority lost one or both parents; thus, educational interventions may be particularly relevant for orphans. Further investigation of these investigational, sociodemographic, and societal factors will improve the targeting and delivery of AGYW’s HIV prevention interventions.

Our analysis focused on individual-level interventions: future research may want to examine the influence of community-level interventions on outcomes, particularly those that may be more directly influenced by community norms and structures (e.g., gender-based violence). We assessed each outcome individually; further analysis maybe warranted for AGYW who reported multiple risk outcomes. Additional analyses should also assess the sustainability of these outcomes over time and the degree of improvement needed for sustainable change. While we investigated optimal intervention combinations, understanding the layered effect of socio-demographic and contextual factors on HIV-related outcomes is also needed. Additionally, the terminal node considered in some of our CART models have very low cell frequencies and, hence, care should be taken when generalizing inference from these nodes. Nevertheless, given the statistical significance of the splits, the results hold true for the study sample. Finally, the current analysis was based on a cross-sectional analysis without a comparison group; thus, the results do not demonstrate causation. It is important to validate the findings with a comparative analysis using longitudinal data to test the causality of these relationships. Overall, we urge further replication of this analysis in other contexts to see if similar combinations/pathways are important for achieving the desired outcomes by age group. It is important to consider these innovative analytical approaches to help program implementers and policy makers make informed decisions about what package of interventions to deliver for AGYW.

## Conclusion

With the proliferation of multi-component programming, insight regarding the optimal combination of HIV prevention interventions is necessary to tailor activities and maximize time, energy, and resources. We examined combinations of health and non-health interventions offered by the DREAMS program in Zambia to assess which intervention permutations led to positive changes in AGYW’s HIV-related outcomes. Exposure to social asset building activities and educational social protections improved AGYW’s HIV-related outcomes. YW benefitted from the provision of youth friendly health services, while economic social protections had effects on AG’s HIV risk. In addition to highlighting the need for differentiated intervention packages, these findings underscore the importance of multi-component programming to reduce AGYW’s HIV risk. Future efforts should consider this program design and delivery model as a viable approach to addressing AGYW’s numerous intersecting HIV vulnerabilities, as it will help AGYW live healthy, fulfilling lives and shift the HIV landscape toward epidemic control.

## Supplementary Material

Supplementary Table 1

Supplementary Figures 1-5

## Figures and Tables

**Table 1.** Definition of study measures

Measure	Coding	Definition
Characteristics		
Orphanhood	Binary (Yes/No)	At least one parent is deceased
Performs any paid work	Binary (Yes/No)	Work/labor is compensated with cash vs. unemployed or is not financially compensated for work.
Ever travelled outside the community	Binary (Yes/No)	Ever moved or travelled/gone elsewhere outside of their community/area
Currently in school	Binary (Yes/No)	Current enrolled in school
DREAMS Interventions		
Exposure to social asset building	Binary (Yes/No)	Completed all 13 safe space group sessions or received a participation certificate signifying completion of the social asset building program.
Received youth-friendly health services	Binary (Yes/No)	Offered at least one SRH service, including HIV testing, condoms, contraceptives/family planning services, pregnancy consultation, STI testing or treatment, post-violence care, or oral pre-exposure prophylaxis through DREAMS.
Received educational social protection	Binary (Yes/No)	Offered money for fees, uniforms, transport, or other help with schooling expenses under DREAMS.
Received economic social protection	Binary (Yes/No)	Offered cash/financial support or participated in the loan and savings club.
Outcomes		
Tested for HIV in the last 12 months	Binary (Yes/No)	Self-reported testing for HIV in the last 12 months
Engagement in transactional sex in the last 12 months (among sexually active)	Binary (Yes/No)	AGYW reported having sex with a man in the last 12 months in expectation to receive or received: a) cash/money to be looked after, b) somewhere to stay, c) support or money for her children or family, d) drugs, food, cosmetics, clothes, a cell phone, airtime, transportation, or anything else she couldn’t afford herself, or e) somewhere to sleep for the night, bills or school fees.
Consistent condom use with the partner in the last 12 months (among sexually active)	Binary (Yes/No)	AGYWs who were in a sexual relationship with their main regular partner or any other partner were asked about frequency of using a condom. The frequency of using a condom was categorized as never, sometimes/inconsistent, always, and don’t know. Those who reported using a condom ‘always’ were described as using condoms consistently.
Sexual violence with intimate partner in the last 12 months (among sexually active)	Binary (Yes/No)	AGYW were asked if their intimate partner had physically forced her to have sex when she did not want to, used threats or intimidation to get her to have sex when she did not want to, and if she was forced to do something else sexually that she did not want to. The response options were never, once, few, many. AGYW who responded never to all above three behaviours from their main partner were defined not to have experienced sexual violence else yes.
Sexual violence with non-intimate partner in the last 12 months	Binary (Yes/No)	All AGYW were asked if a man who is not their boyfriend or partner had forced or persuaded her to have sex against her will, forced or persuaded to have sex against her will and did not succeed, and forced her to have sex against her will when she was too inebriated (due to alcohol or drugs) to refuse. The response options were never, once, few, many. AGYWs who responded never to all of the above five experiences were defined not to have experienced sexual violence with a non-intimate partner else yes.

**Table 2.** Profile of adolescent girls (aged 15–19 years) and young women (aged 20–25 years), by age group, March-May 2018

Characteristics	Overall (N=885)	15–19 years old (N=380)	20–25 years old (N=505)

	%	%	%

Orphan	41.1	36.1	45.0
Does any paid work	45.0	49.2	41.8
Ever travelled outside of this community	75.4	72.1	77.8
Currently in school	56.8	66.3	49.7
Social asset building (SAB)	59.9	62.6	57.8
Youth friendly health services (YFHS)	81.5	86.6	77.6
Educational social protection (Educ)	35.4	48.9	25.1
Economic social protection (Econ)	29.6	32.9	27.1

**Table 3:** Predicting HIV testing, consistent condom use, no transactional sex, and no experience of sexual violence among adolescent girls (AG, aged 15–19 years) and young women (YW, aged 20–25 years) using Classification and Regression Trees

HIV-related Outcomes	**Intervention Components** ^†^				Base value^‡^ (%)	% Increase from base value^§^
	SAB	YFHS	Educ	Econ		
***HIV testing***						
AG	Yes	Yes	Yes	Yes	72.6	14.7
YW	No		Yes	No	76.8	18.4
***Consistent condom use***						
AG				Yes	33.3	38.7
YW	Yes	Yes	No	No	35.6	46.1
***No transactional sex***						
AG						
Pathway 1	No	No			93.8	6.6
Pathway 2	Yes		Yes		93.8	6.6
YW						
Pathway 1		No		Yes	95.3	4.9
Pathway 2	No	Yes	Yes	Yes	95.3	4.9
Pathway 3	Yes	Yes	Yes	No	95.3	4.9
***No intimate partner sexual violence***						
AG			Yes	No	76.3	9.2
YW	No	Yes		No	81.6	7.1
***No non-partner sexual violence***						
AG	Yes	No	No	No	88.7	12.7
YW	Yes		Yes	No	86.9	10.8

† Intervention components: SAB = Social Asset Building, YFHS = Youth Friendly sexual and reproductive Health Services, Educ = Educational social protection, Econ = Economic social protection

‡ Base value here refers to the prevalence in the sample. The CART analysis shows the likelihood of change from this value given different exposures.

§ % increase from base value refers to percentage improvement in prevalence of an outcome attributed to the pathway.

Interpretation: Yes, indicates that the AG received the intervention component, No indicates that they did not, and a blank cell indicates that the intervention component did not feature in any of the optimal pathways in the CART model.

## Data Availability

The underlying data are available on Dataverse.^57^
