# Supplementary material for: Which intervention synergies maximize AGYW’s HIV outcomes? A classification and regression tree analysis of layered HIV prevention programming
Source: J Acquir Immune Defic Syndr. Author manuscript; Available in PMC 2024 Dec 1. (PMC10617659; doi:10.1097/QAI.0000000000003289)
Supplement: Supplementary Table 1 [file NIHMS1925538-supplement-Supplementary_Table_1.docx]

**Supplemental Table 1.** Characteristics of adolescent girls (aged 15-19 years) and young women (aged 20-25 years) in each pathway

|  | **Age category** | **Pathway** | **Orphanhood** | **Paid work** | **Ever travelled outside community** | **Currently enrolled in school** |
| --- | --- | --- | --- | --- | --- | --- |
| **Tested for HIV in last 12 months** | 15-19 | Pathway 1 | 25.6% | 14.9% | 67.9% | 69.2% |
|  | 20-25 | Pathway 1 | 54.5% | 62.5% | 81.8% | 9.1% |
| **Consistent condom** | 15-19 | Pathway 1 | 30.4% | 18.6% | 68.8% | 66.4% |
|  | 20-25 | Pathway 1 | 40.8% | 67.8% | 76.0% | 64.0% |
| **No transactional sex** | 15-19 | Pathway 1 | 39.5% | 28.6% | 74.4% | 74.4% |
|  | 15-19 | Pathway 2 | 26.9% | 16.2% | 69.2% | 69.2% |
|  | 20-25 | Pathway 1 | 40.0% | 75.0% | 100% | 40.0% |
|  | 20-25 | Pathway 2 | 50.0% | 60.0% | 92.3% | 30.8% |
|  | 20-25 | Pathway 3 | 53.3% | 60.0% | 80.0% | 40.0% |
| **No Sexual violence from partner** | 15-19 | Pathway 1 | 40.4% | 26.3% | 76.4% | 66.3% |
|  | 20-25 | Pathway 1 | 44.0% | 69.2% | 80.7% | 40.4% |
| **No sexual violence from non-partner** | 15-19 | Pathway 1 | 41.0% | 33.3% | 76.9% | 71.8% |
|  | 20-25 | Pathway 1 | 51.9% | 63.6% | 88.9% | 33.3% |
