# Supplementary material for: Which intervention synergies maximize AGYW’s HIV outcomes? A classification and regression tree analysis of layered HIV prevention programming
Source: J Acquir Immune Defic Syndr. Author manuscript; Available in PMC 2024 Dec 1. (PMC10617659; doi:10.1097/QAI.0000000000003289)
Supplement: Supplementary Figures 1-5 [file NIHMS1925538-supplement-Supplementary_Figures_1-5.pptx]

## Slide 1
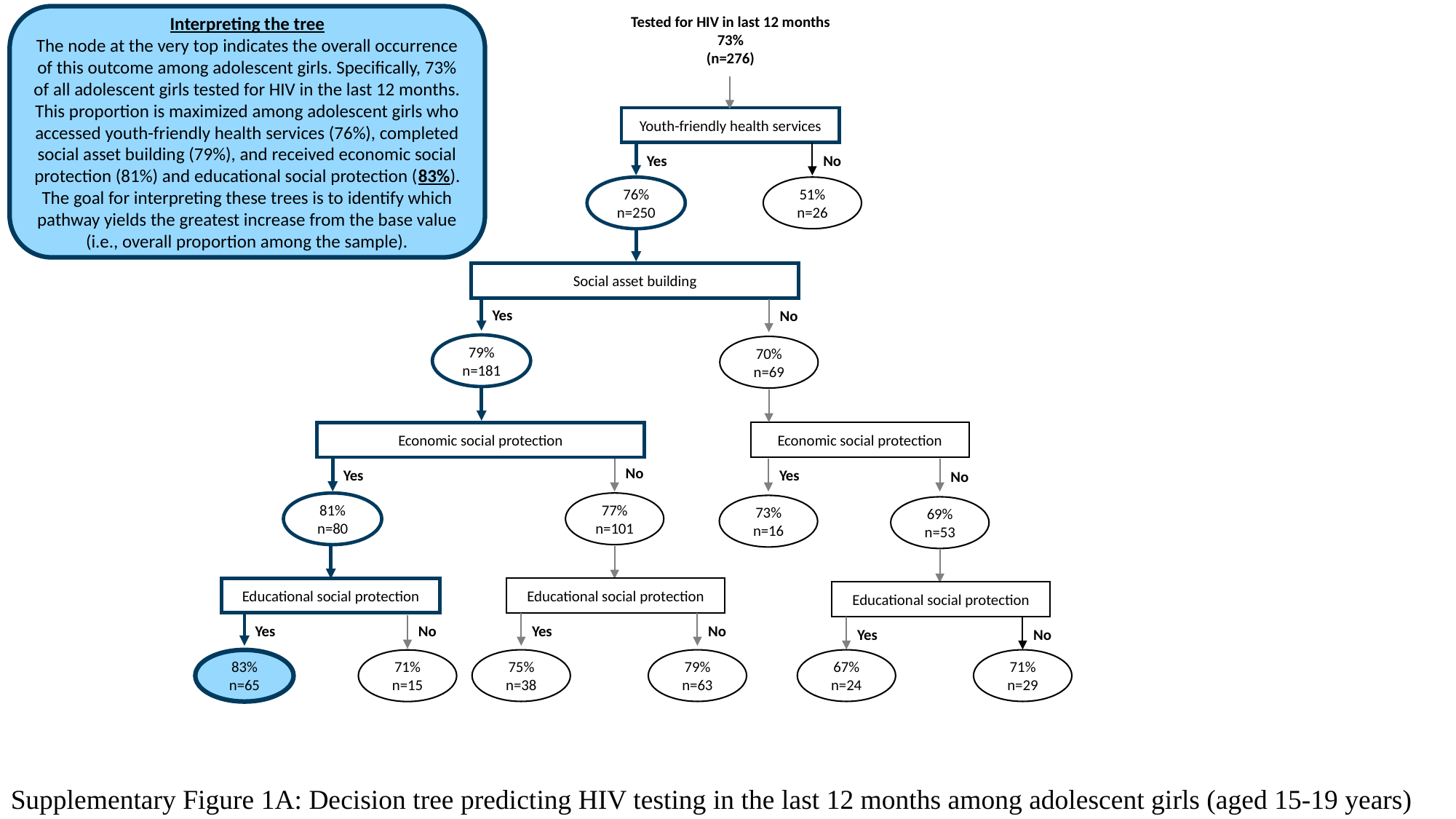

Interpreting the tree
The node at the very top indicates the overall occurrence of this outcome among adolescent girls. Specifically, 73% of all adolescent girls tested for HIV in the last 12 months. This proportion is maximized among adolescent girls who accessed youth-friendly health services (76%), completed social asset building (79%), and received economic social protection (81%) and educational social protection (83%). The goal for interpreting these trees is to identify which pathway yields the greatest increase from the base value (i.e., overall proportion among the sample).
Tested for HIV in last 12 months
73%
(n=276)
Youth-friendly health services
Yes
No
76%
n=250
51%
n=26
Social asset building
Yes
No
79%
n=181
70%
n=69
Economic social protection
Economic social protection
No
Yes
Yes
No
81%
n=80
77%
n=101
73%
n=16
69%
n=53
Educational social protection
Educational social protection
Educational social protection
Yes
No
Yes
No
Yes
No
75%
n=38
79%
n=63
67%
n=24
71%
n=29
83%
n=65
71%
n=15
Supplementary Figure 1A: Decision tree predicting HIV testing in the last 12 months among adolescent girls (aged 15-19 years)

## Slide 2
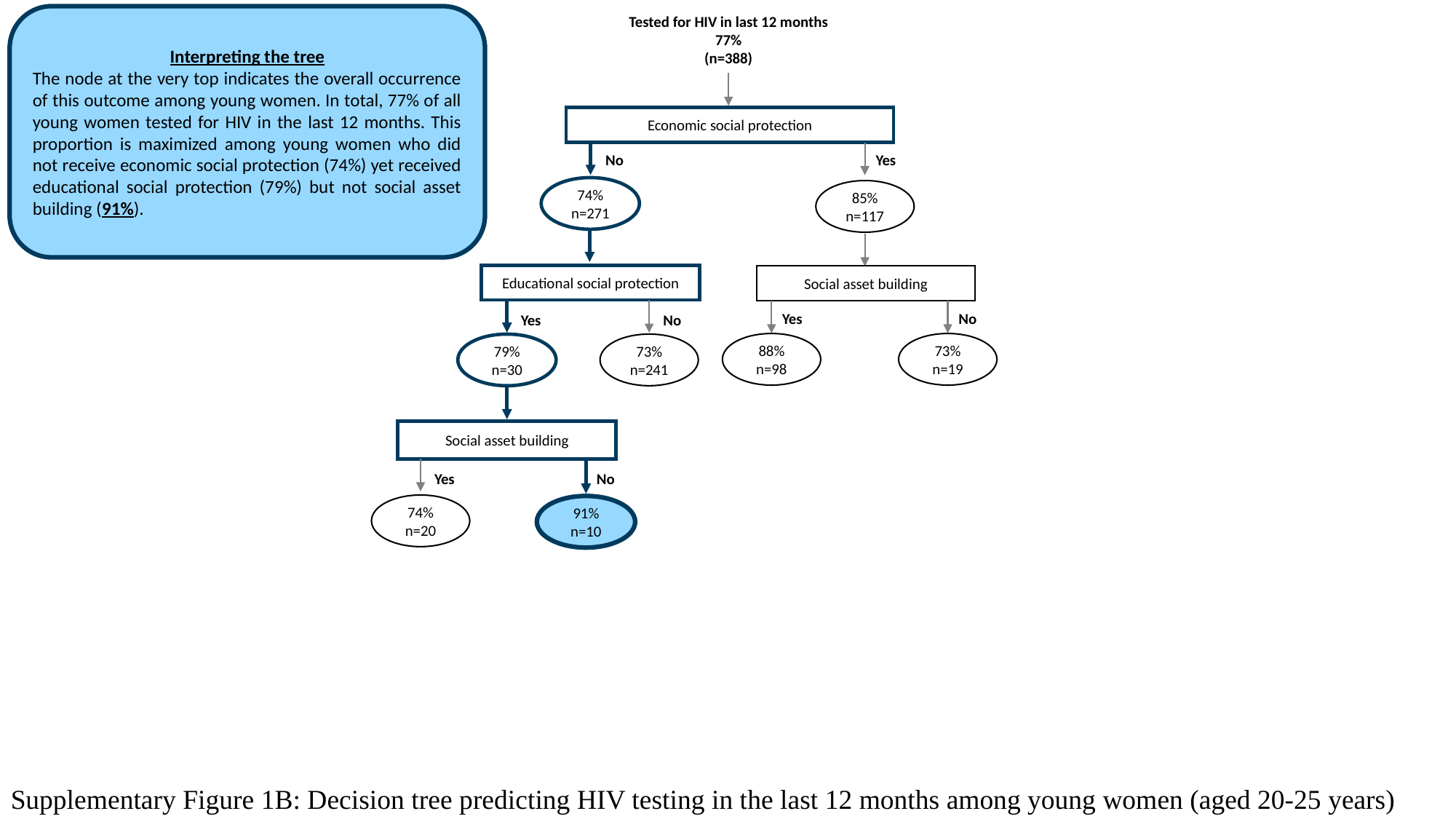

Interpreting the tree
The node at the very top indicates the overall occurrence of this outcome among young women. In total, 77% of all young women tested for HIV in the last 12 months. This proportion is maximized among young women who did not receive economic social protection (74%) yet received educational social protection (79%) but not social asset building (91%).
Tested for HIV in last 12 months
77%
(n=388)
Economic social protection
No
Yes
74%
n=271
85%
n=117
Educational social protection
Social asset building
Yes
No
Yes
No
88%
n=98
73%
n=19
79%
n=30
73%
n=241
Social asset building
Yes
No
74%
n=20
91%
n=10
Supplementary Figure 1B: Decision tree predicting HIV testing in the last 12 months among young women (aged 20-25 years)

## Slide 3
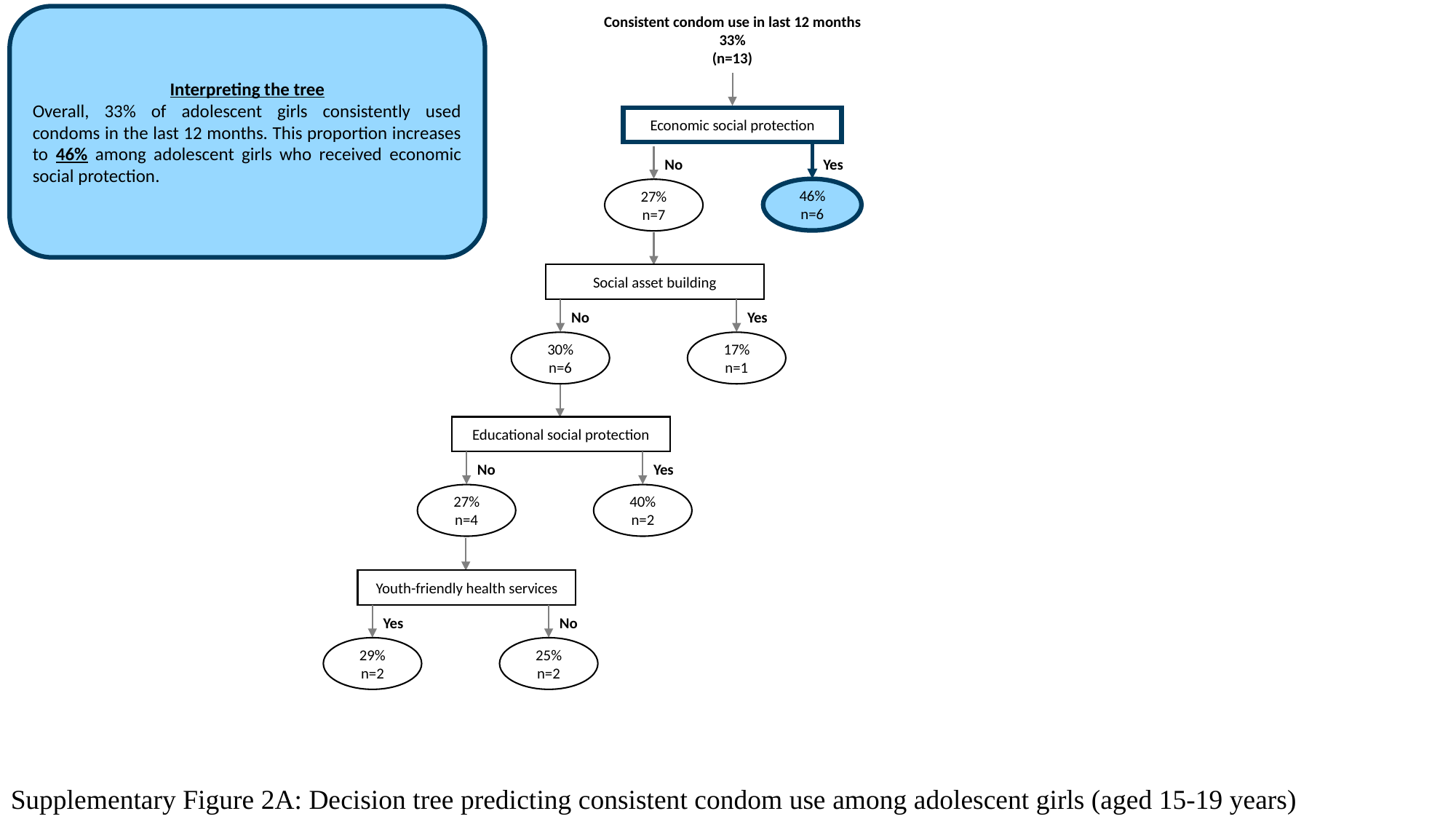

Interpreting the tree
Overall, 33% of adolescent girls consistently used condoms in the last 12 months. This proportion increases to 46% among adolescent girls who received economic social protection.
Consistent condom use in last 12 months 33%
(n=13)
Economic social protection
No
Yes
46%
n=6
27%
n=7
Social asset building
No
Yes
30%
n=6
17%
n=1
Educational social protection
No
Yes
27%
n=4
40%
n=2
Youth-friendly health services
Yes
No
29%
n=2
25%
n=2
Supplementary Figure 2A: Decision tree predicting consistent condom use among adolescent girls (aged 15-19 years)

## Slide 4
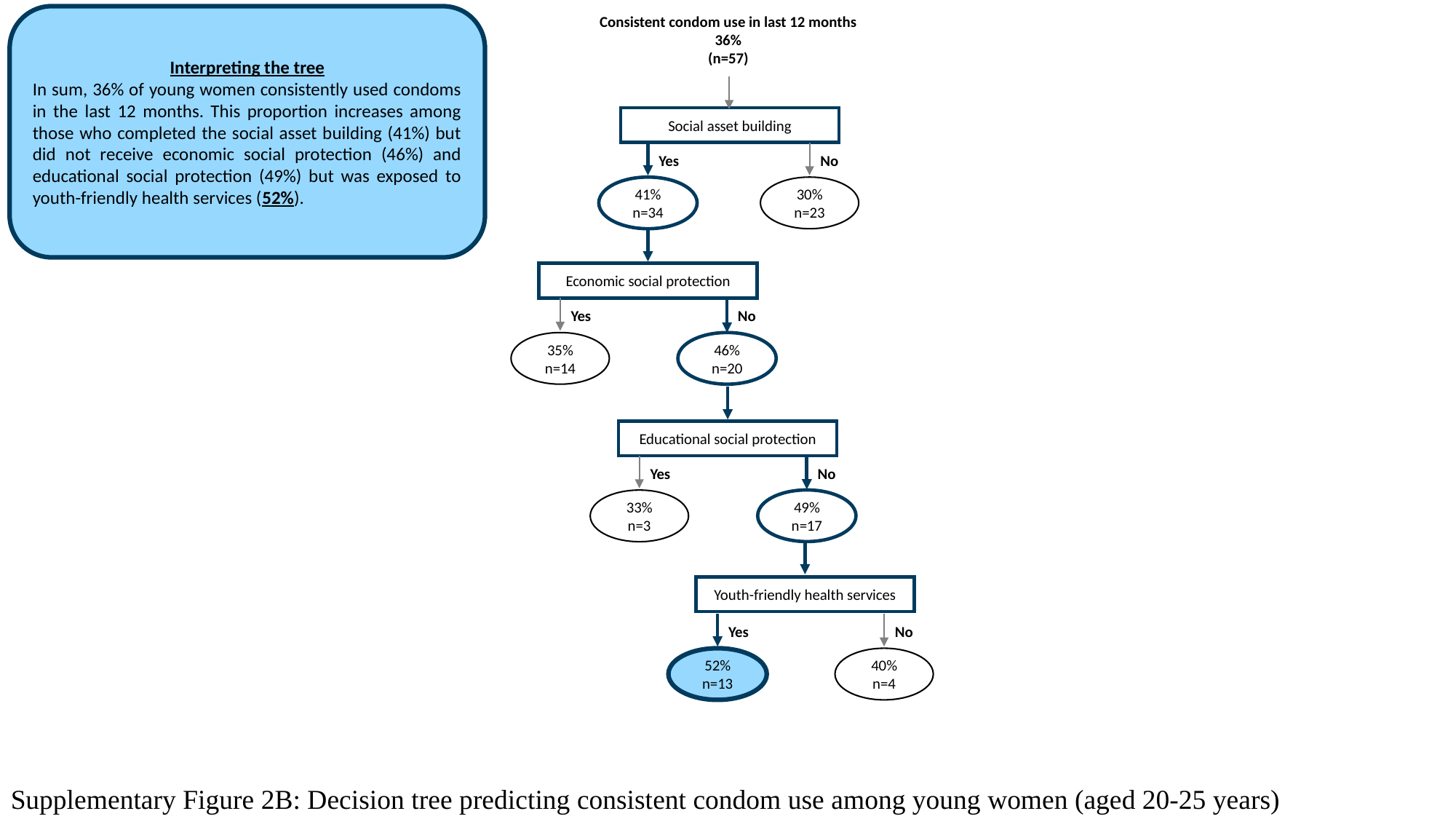

Interpreting the tree
In sum, 36% of young women consistently used condoms in the last 12 months. This proportion increases among those who completed the social asset building (41%) but did not receive economic social protection (46%) and educational social protection (49%) but was exposed to youth-friendly health services (52%).
Consistent condom use in last 12 months 36%
(n=57)
Social asset building
Yes
No
41%
n=34
30%
n=23
Economic social protection
Yes
No
35%
n=14
46%
n=20
Educational social protection
Yes
No
49%
n=17
33%
n=3
Youth-friendly health services
No
Yes
52%
n=13
40%
n=4
Supplementary Figure 2B: Decision tree predicting consistent condom use among young women (aged 20-25 years)

## Slide 5
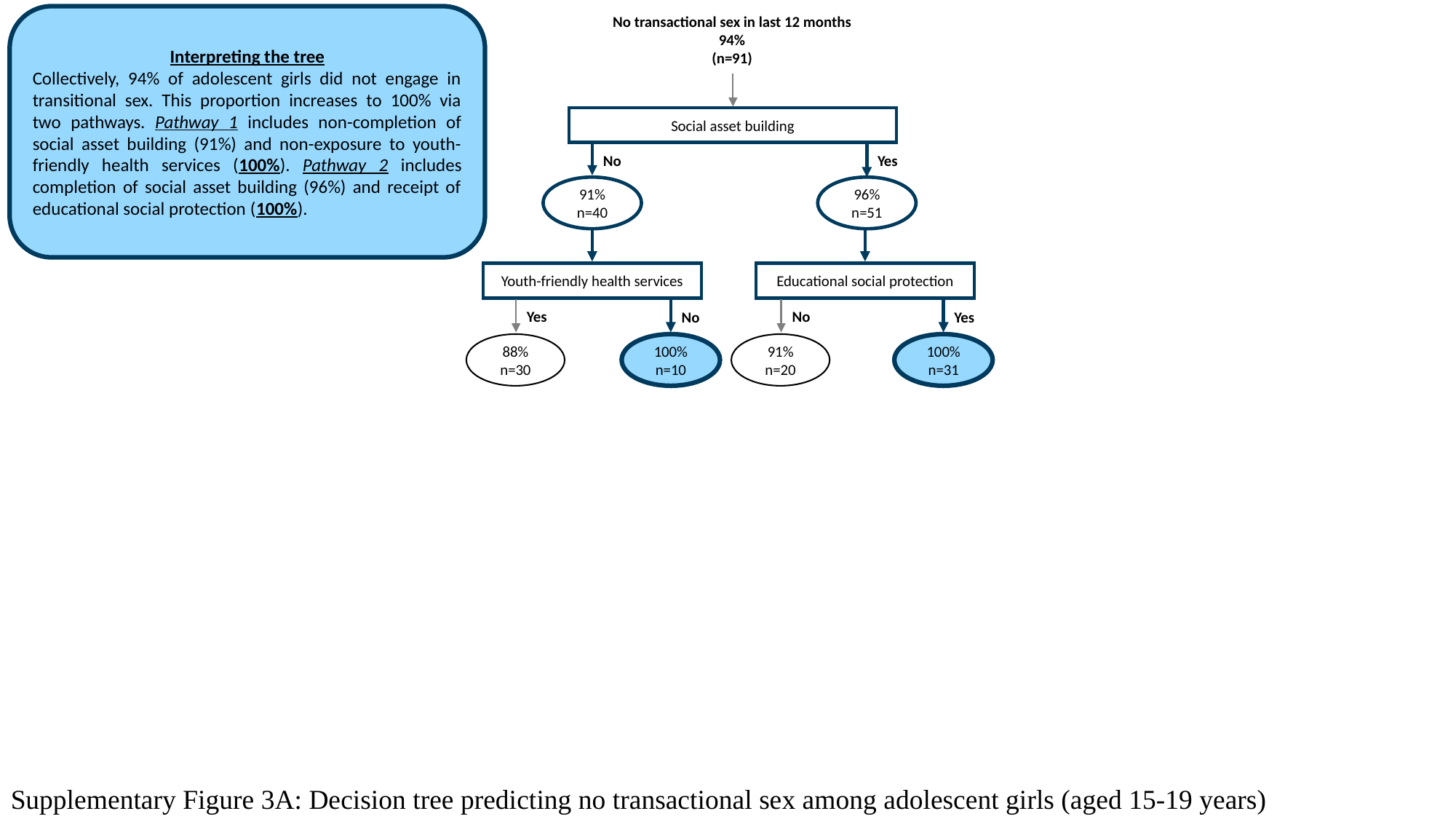

Interpreting the tree
Collectively, 94% of adolescent girls did not engage in transitional sex. This proportion increases to 100% via two pathways. Pathway 1 includes non-completion of social asset building (91%) and non-exposure to youth-friendly health services (100%). Pathway 2 includes completion of social asset building (96%) and receipt of educational social protection (100%).
No transactional sex in last 12 months
94%
(n=91)
Social asset building
No
Yes
91%
n=40
96%
n=51
Youth-friendly health services
Educational social protection
Yes
No
Yes
No
88%
n=30
100%
n=10
91%
n=20
100%
n=31
Supplementary Figure 3A: Decision tree predicting no transactional sex among adolescent girls (aged 15-19 years)

## Slide 6
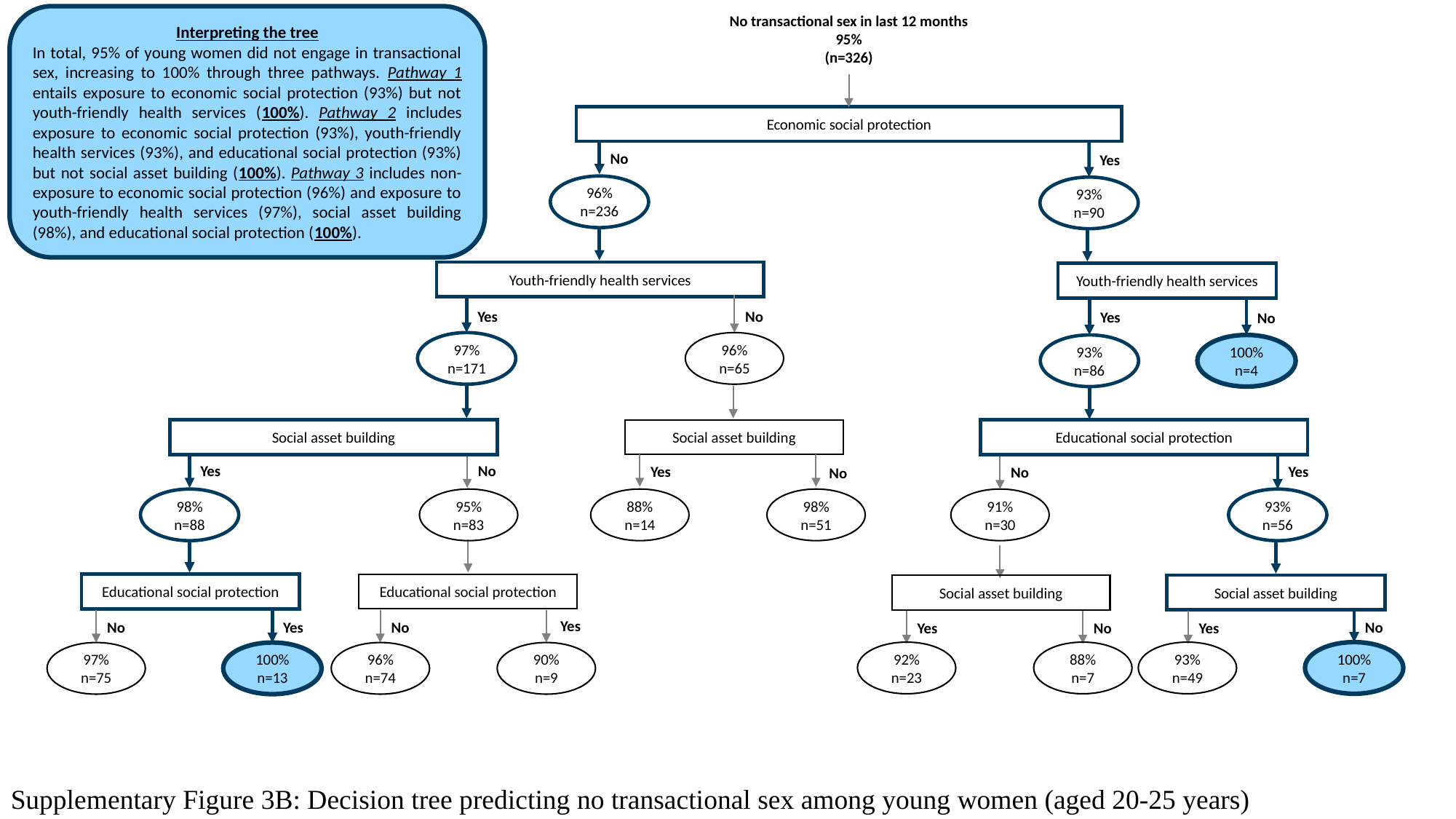

No transactional sex in last 12 months
95%
(n=326)
Interpreting the tree
In total, 95% of young women did not engage in transactional sex, increasing to 100% through three pathways. Pathway 1 entails exposure to economic social protection (93%) but not youth-friendly health services (100%). Pathway 2 includes exposure to economic social protection (93%), youth-friendly health services (93%), and educational social protection (93%) but not social asset building (100%). Pathway 3 includes non-exposure to economic social protection (96%) and exposure to youth-friendly health services (97%), social asset building (98%), and educational social protection (100%).
Economic social protection
No
Yes
96%
n=236
93%
n=90
Youth-friendly health services
Youth-friendly health services
Yes
No
Yes
No
97%
n=171
96%
n=65
93%
n=86
100%
n=4
Social asset building
Social asset building
Educational social protection
No
Yes
Yes
Yes
No
No
98%
n=88
95%
n=83
88%
n=14
98%
n=51
91%
n=30
93%
n=56
Educational social protection
Educational social protection
Social asset building
Social asset building
Yes
No
Yes
No
No
Yes
No
Yes
92%
n=23
88%
n=7
93%
n=49
100%
n=7
97%
n=75
100%
n=13
96%
n=74
90%
n=9
Supplementary Figure 3B: Decision tree predicting no transactional sex among young women (aged 20-25 years)

## Slide 7
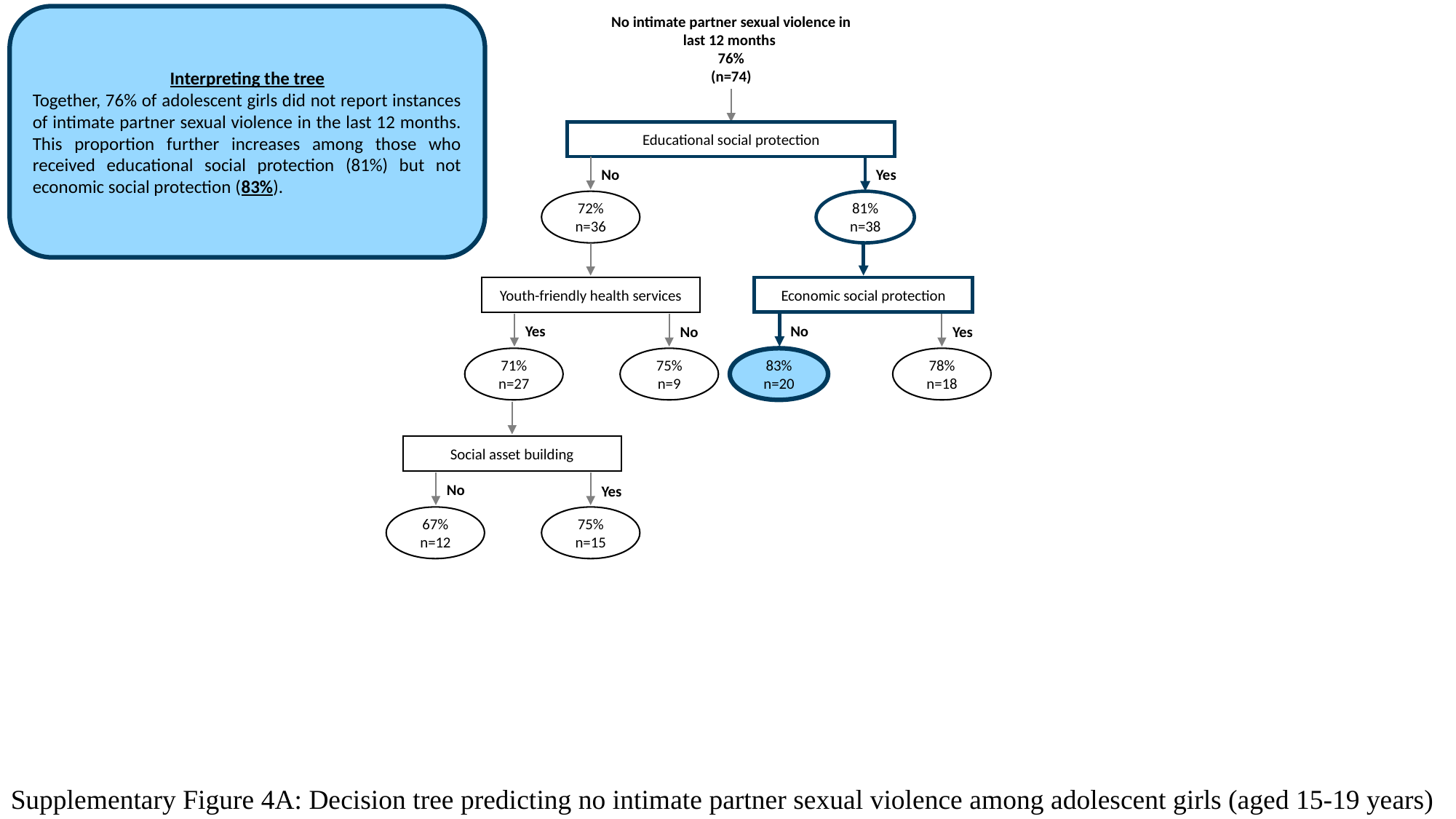

Interpreting the tree
Together, 76% of adolescent girls did not report instances of intimate partner sexual violence in the last 12 months. This proportion further increases among those who received educational social protection (81%) but not economic social protection (83%).
No intimate partner sexual violence in last 12 months
76%
(n=74)
Educational social protection
No
Yes
72%
n=36
81%
n=38
Youth-friendly health services
Economic social protection
Yes
No
Yes
No
71%
n=27
75%
n=9
83%
n=20
78%
n=18
Social asset building
No
Yes
67%
n=12
75%
n=15
Supplementary Figure 4A: Decision tree predicting no intimate partner sexual violence among adolescent girls (aged 15-19 years)

## Slide 8
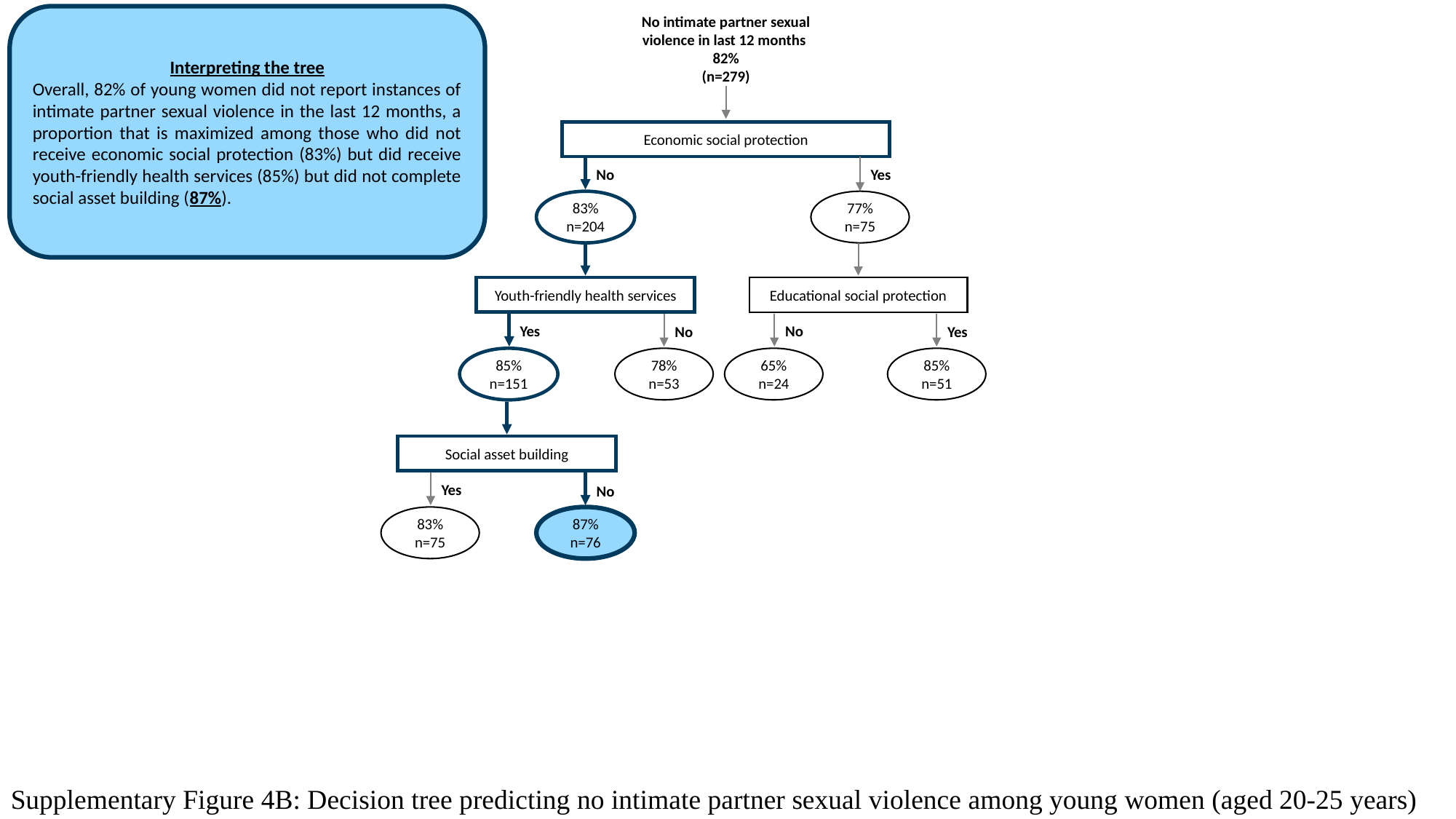

Interpreting the tree
Overall, 82% of young women did not report instances of intimate partner sexual violence in the last 12 months, a proportion that is maximized among those who did not receive economic social protection (83%) but did receive youth-friendly health services (85%) but did not complete social asset building (87%).
No intimate partner sexual violence in last 12 months
82%
(n=279)
Economic social protection
No
Yes
83%
n=204
77%
n=75
Youth-friendly health services
Educational social protection
Yes
No
Yes
No
85%
n=151
78%
n=53
65%
n=24
85%
n=51
Social asset building
Yes
No
83%
n=75
87%
n=76
Supplementary Figure 4B: Decision tree predicting no intimate partner sexual violence among young women (aged 20-25 years)

## Slide 9
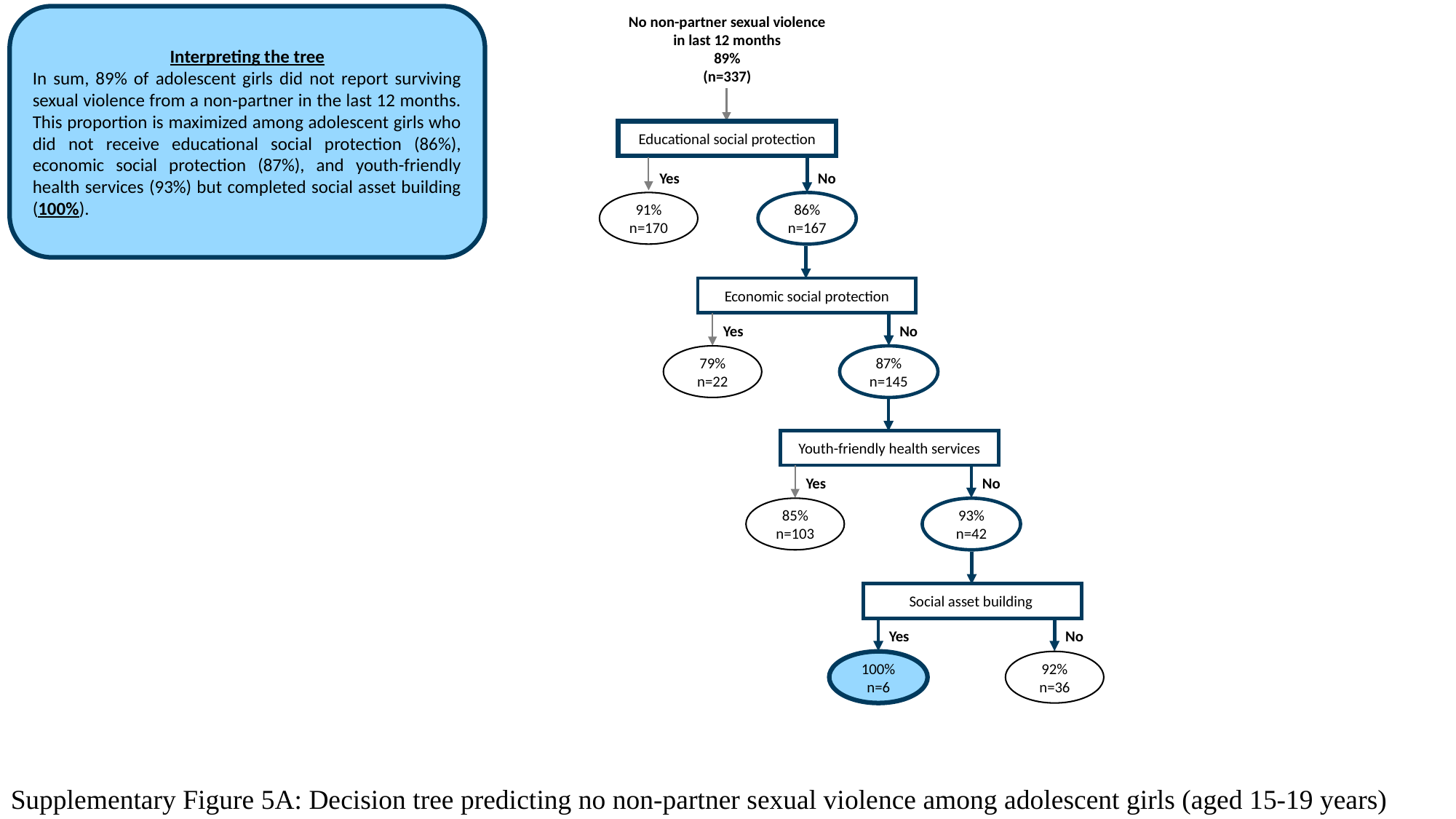

Interpreting the tree
In sum, 89% of adolescent girls did not report surviving sexual violence from a non-partner in the last 12 months. This proportion is maximized among adolescent girls who did not receive educational social protection (86%), economic social protection (87%), and youth-friendly health services (93%) but completed social asset building (100%).
No non-partner sexual violence in last 12 months
89%
(n=337)
Educational social protection
Yes
No
91%
n=170
86%
n=167
Economic social protection
Yes
No
79%
n=22
87%
n=145
Youth-friendly health services
Yes
No
85%
n=103
93%
n=42
Social asset building
Yes
No
100%
n=6
92%
n=36
Supplementary Figure 5A: Decision tree predicting no non-partner sexual violence among adolescent girls (aged 15-19 years)

## Slide 10
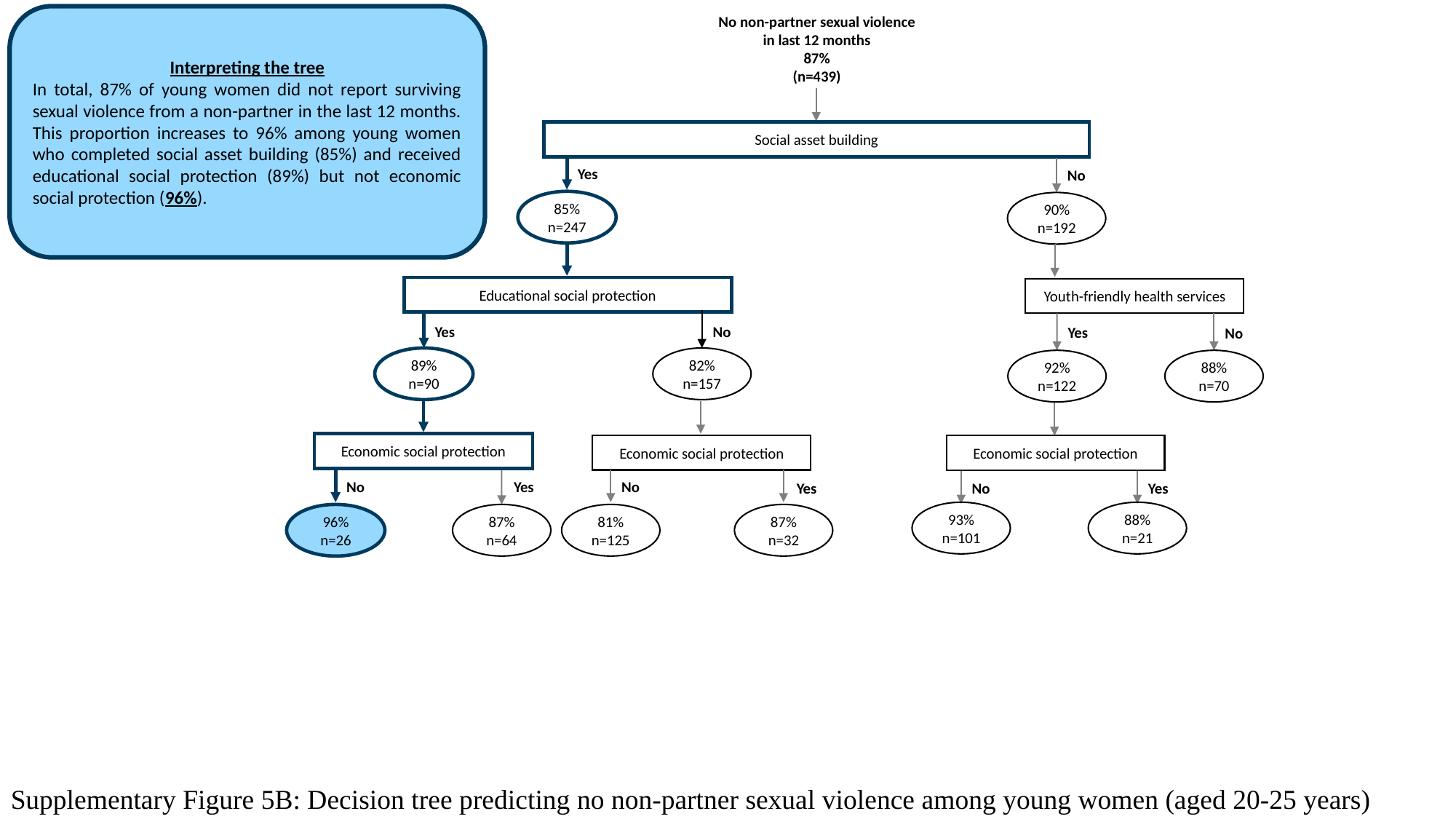

Interpreting the tree
In total, 87% of young women did not report surviving sexual violence from a non-partner in the last 12 months. This proportion increases to 96% among young women who completed social asset building (85%) and received educational social protection (89%) but not economic social protection (96%).
No non-partner sexual violence in last 12 months
87%
(n=439)
Social asset building
Yes
No
85%
n=247
90%
n=192
Educational social protection
Youth-friendly health services
Yes
No
Yes
No
89%
n=90
82%
n=157
92%
n=122
88%
n=70
Economic social protection
Economic social protection
Economic social protection
Yes
No
No
Yes
No
Yes
93%
n=101
88%
n=21
96%
n=26
87%
n=64
81%
n=125
87%
n=32
Supplementary Figure 5B: Decision tree predicting no non-partner sexual violence among young women (aged 20-25 years)
